# Role of STAR and SCP2/SCPx in the Transport of Cholesterol and Other Lipids

**DOI:** 10.3390/ijms232012115

**Published:** 2022-10-11

**Authors:** Melanie Galano, Sathvika Venugopal, Vassilios Papadopoulos

**Affiliations:** 1 Department of Pharmacology and Pharmaceutical Sciences, School of Pharmacy, University of Southern California, Los Angeles, CA 90089, USA; 2 Department of Medicine, The Research Institute of the McGill University Health Centre, McGill University, Montreal, QC H4A 3J1, Canada

**Keywords:** cholesterol, cholesterol transport, steroidogenic acute regulatory protein, sterol carrier protein 2, sterol carrier protein-x

## Abstract

Cholesterol is a lipid molecule essential for several key cellular processes including steroidogenesis. As such, the trafficking and distribution of cholesterol is tightly regulated by various pathways that include vesicular and non-vesicular mechanisms. One non-vesicular mechanism is the binding of cholesterol to cholesterol transport proteins, which facilitate the movement of cholesterol between cellular membranes. Classic examples of cholesterol transport proteins are the steroidogenic acute regulatory protein (STAR; STARD1), which facilitates cholesterol transport for acute steroidogenesis in mitochondria, and sterol carrier protein 2/sterol carrier protein-x (SCP2/SCPx), which are non-specific lipid transfer proteins involved in the transport and metabolism of many lipids including cholesterol between several cellular compartments. This review discusses the roles of STAR and SCP2/SCPx in cholesterol transport as model cholesterol transport proteins, as well as more recent findings that support the role of these proteins in the transport and/or metabolism of other lipids.

## 1. Introduction

Cholesterol is a lipid molecule that has been associated with many diseases such as cardiovascular disease, atherosclerosis, Alzheimer’s disease, and different types of cancers [1,2,3]. While much of the cholesterol research has focused on its dysregulation and role in the pathology of these diseases, it is also an essential lipid molecule critical to the maintenance of a variety of indispensable functions and pathways. Cholesterol is a 27-carbon molecule containing four fused rings and an 8-carbon tail, making it a hydrophobic molecule [4]. As such, cholesterol is a major component of cellular membranes, where cholesterol interacts with other lipids to regulate the membrane permeability of certain ions and solutes, the rigidity of the membrane to act as a scaffold for membrane proteins, and the fluidity of the membrane for rapid diffusion, budding to form vesicles, or fusion with other membranes for trafficking [5]. Furthermore, cholesterol is the precursor of all steroid hormones, which play vital roles in reproduction, salt and water balance, and stress response [4]. It is also metabolized into various oxysterols that then give rise to bile acids, which are critical for the absorption and digestion of lipids [4,6]. Because of its role in several key cellular processes, cholesterol must be properly trafficked throughout the cell, which occurs via both vesicular and non-vesicular pathways, of which the latter involves cholesterol transport proteins [7]. Cholesterol interacts with a variety of cholesterol transport proteins for its intracellular trafficking, such as the steroidogenic acute regulatory protein (STAR; STARD1), a key protein in acute steroidogenesis, and sterol carrier protein 2/sterol carrier protein-x (SCP2/SCPx), which are non-specific lipid transport proteins that may play roles in cholesterol transport and metabolism [8,9]. While STAR is classically known to transport cholesterol for mitochondrial steroidogenesis, recent data suggest that STAR may also function in the transport and/or metabolism of other lipids [10]. Additionally, since the discovery that STAR and not SCP2 is the key cholesterol transporter for steroid biosynthesis, the general role of SCP2/SCPx in the transport of cholesterol and other lipids has been ignored [11,12,13]. In this review, we discuss intracellular cholesterol distribution and current evidence for the multifunctional roles of STAR and SCP2/SCPx as model cholesterol transport proteins.

### 1.1. Synthesis

Cholesterol is derived from two sources: de novo synthesis or dietary intake. While all mammalian cells are able to synthesize cholesterol, steroidogenic cells of the adrenals, testis, ovaries, and brain synthesize cholesterol at the highest rates [14,15]. De novo cholesterol biosynthesis occurs in the endoplasmic reticulum (ER) by a complex process involving several, tightly regulated enzymes of the mevalonate pathway [4,16,17].

In addition to de novo cholesterol synthesis, cholesterol is also supplied to the cell via dietary intake. Cholesterol derived from food is absorbed by the small intestine, packed into chylomicrons, and transported to the liver, where cholesterol is processed into very low-density lipoproteins (VLDLs) [18]. Through circulation, VLDLs become low-density lipoproteins (LDLs), which bind to LDL receptors (LDLR) on the cell surface. These complexes are then endocytosed through clathrin-coated pits into the cytoplasm, which then fuse to endosomal compartments for processing the LDLs, while LDLR is recycled back to the cell surface. The cholesteryl esters derived from LDLs are subsequently de-esterified to free cholesterol by lysosomal acid lipase [4,19]. In contrast, another mechanism of cholesterol uptake into the cell occurs via a non-endocytic pathway involving the uptake of high-density lipoproteins (HDLs) by scavenger receptor class B, type I (SR-BI), which is mostly expressed in steroidogenic cells of the testes, ovaries, and adrenals [20]. HDL particles bind SR-BI, which transfers HDL cholesterol to the plasma membrane [21]. The cholesteryl esters derived from HDLs are then hydrolyzed by hormone-sensitive lipase, forming free cholesterol [22].

### 1.2. Distribution

Regulation of cellular cholesterol levels occurs at many levels. Cholesterol biosynthesis can be regulated by the sterol regulatory element-binding protein 2, which regulates transcription of genes important for cholesterol biosynthesis, and by HMG-CoaA reductase enzyme (HMGCR) [1]. Additionally, cholesterol homeostasis may be regulated by controlling LDLR-mediated uptake or by controlling cholesterol efflux by cholesterol efflux transporters such as ATP-binding cassette subfamily A member 1 [1]. Further, levels of cholesterol can be controlled by cholesterol esterification through the regulation of acyl-coenzyme A: cholesterol acyltransferases (ACAT1 and ACAT2) [23]. While active, free cholesterol is most commonly found in membranes or trafficked to other organelles for other functions, cholesterol esterification prevents the accumulation of free cholesterol and primes esterified cholesterol for storage in lipid droplets [24].

Lipids in biological mammalian membranes include sphingolipids, glycerol-based lipids, and cholesterol, and the lipid composition of a specific membrane is determined in part by its subcellular location [25]. In the cell, about 65%–80% of cellular cholesterol is present at the plasma membrane, while the Golgi apparatus and endosomal recycling compartment (ERC), which are closely associated with the plasma membrane, have intermediate cholesterol levels [26]. However, the ER has only 0.1%–2% of total cellular cholesterol despite this being the site of cholesterol biosynthesis [27]. This has led to the notion that newly synthesized cholesterol in the ER is rapidly transported to other organelles for other functions or stored in lipid droplets as cholesteryl esters. Additionally, mitochondria are also cholesterol-poor, despite mitochondria being the initiation site of steroidogenesis [28,29]. Therefore, these low intrinsic concentrations necessitate the rapid transport of cholesterol from intracellular stores into mitochondria for steroid production [30].

### 1.3. Intracellular Trafficking: Vesicular and Non-Vesicular Cholesterol Transport

The transport of cholesterol between organelles or from intracellular stores is vital for many cellular processes; therefore, proper cholesterol trafficking is finely regulated. Because cholesterol itself is insoluble, it is trafficked in the cell in two general ways: vesicular pathways and non-vesicular pathways. Vesicular cholesterol transport involves the delivery of membrane cholesterol via a vesicle formed by budding off of the donor membrane, followed by vesicle fusion with another membrane [31]. Vesicular cholesterol transport, such as the trafficking of cholesterol via internalization of LDL-derived cholesterol, vesicle formation, and movement of cholesterol through the endosomal and lysosomal pathways, requires an intact cytoskeleton and metabolic energy [27,32].

Although much work has been done elucidating mechanisms of vesicular cholesterol trafficking, evidence shows that inhibiting vesicular transport by genetic or pharmacological means does not inhibit intracellular cholesterol transport [33,34,35]. Furthermore, work has shown that cholesterol trafficking between the ER and plasma membrane occurs much faster than vesicular trafficking via membrane proteins [36], implying robust non-vesicular trafficking. Non-vesicular cholesterol trafficking involves the transport of cholesterol from a donor membrane to an acceptor membrane that does not require metabolic energy. This process can occur spontaneously via passive diffusion, the rate of which is dependent on the donor membrane’s lipid composition; by cholesterol-transport proteins, either soluble or membrane-bound; or by membrane contact sites that involve brief interactions between membranes [37]. Although passive diffusion of cholesterol is a slow process, cholesterol-transport proteins or membrane-contact sites can accelerate cholesterol transport [32,37]. This review focuses on the role of cholesterol-transport proteins in intracellular cholesterol trafficking for various cellular processes.

### 1.4. Cholesterol Transport in Steroid Biosynthesis

One important transport route of cholesterol is the delivery of cholesterol from intracellular stores into mitochondria for steroid biosynthesis. The rate-limiting step of acute steroid biosynthesis is the transport of cholesterol derived from intracellular stores at the outer mitochondrial membrane (OMM) to the matrix side of the IMM where cytochrome P450 side chain cleavage enzyme CYP11A1 resides [38,39,40]. This process is initiated by stimulation of steroidogenic cells by the pituitary trophic hormones, luteinizing hormone (LH), follicle stimulating hormone (FSH), and adrenocorticotropic hormone (ACTH), which induce cyclic AMP (cAMP) production [41]. Previous work in our laboratory showed that hormonal stimulation and cAMP induction initiates the formation of a multi-protein complex called the transduceosome, consisting of cytosolic and OMM proteins that together transport cholesterol across the OMM [42]. The cytosolic components of the transduceosome include acyl-CoA binding domain-containing 3, protein kinase A regulatory subunit 1, and hormone-induced STAR, which are anchored to TSPO and voltage dependent anion channel 1 (VDAC1), the OMM components of the transduceosome. While the exact mechanism by which cholesterol moves through the transduceosome is still unclear, disruption of the interactions between these proteins and knockdown or deletion studies have demonstrated the importance of each of these proteins in cholesterol transport for steroid biosynthesis [10,42,43,44,45]. Once at the OMM, proteins of the steroidogenic metabolon, which include TSPO and VDAC along with the IMM proteins ATPase family AAA domain-containing protein 3A and CYP11A1, facilitate the transport of cholesterol to the matrix side of the IMM, where cholesterol is converted to pregnenolone by CYP11A1 [46].

## 2. Model Cholesterol-Transport Proteins

There are several proteins and protein families that facilitate non-vesicular cholesterol transport between membranes. Here, we discuss STAR, which is critical for cholesterol transport into the mitochondria for hormone-induced steroidogenesis, and SCP2/SCPx, which are non-specific lipid transfer proteins that play a role in the transport and/or metabolism of many lipids including cholesterol, as model cholesterol-transport proteins.

### 2.1. STAR

Hormonal stimulation of steroidogenic cells results in the rapid delivery of cholesterol, the precursor of all steroids, to the IMM where cholesterol is converted to pregnenolone, the first step in steroidogenesis [38]. One of the proteins indispensable for steroid biosynthesis is STAR, which is known to facilitate the transfer of cholesterol to mitochondria upon hormonal stimulation [47]. In addition to STAR, there are many other proteins and enzymes involved in steroid biosynthesis, spanning several cellular compartments. The direct interaction between STAR and several of these proteins was shown to be critical for steroidogenesis [30,48].

#### 2.1.1. Role of STAR in Steroidogenesis

Because steroidogenic cells store only very low amounts, steroids must be synthesized rapidly in response to hormonal stimulation. While basal steroidogenesis involves the slow process of transcribing steroidogenic enzymes, hormone-stimulated steroidogenesis involves the rapid transfer of cholesterol to CYP11A1 [39,40,49]. STAR was first discovered when it was shown that acute steroidogenic responses paralleled the synthesis of a 37 kDa phosphoprotein, steroidogenic acute regulatory protein or STAR [50,51]. Overexpression of STAR in MA-10 mouse tumor Leydig cells was found to induce steroidogenesis to a similar extent as cAMP, and introduction of STAR into non-steroidogenic COS-1 cells transfected with the CYP11A1 system increased steroidogenesis 6-fold [47,52,53,54]. Furthermore, the key role of STAR in cholesterol transport for acute steroidogenesis was shown when STAR mutations in humans caused congenital lipoid adrenal hyperplasia (lipoid CAH), a disease characterized by severe deficiency in steroid production and accumulation of cholesterol in steroidogenic cells [55,56]. *Star* knockout (KO) in mice has a similar phenotype as humans with *STAR* mutations; however, gonadal function was less affected in mice [57].

#### 2.1.2. STAR Protein Activity

STAR is synthesized as a 37 kDa cytosolic preprotein composed of an N-terminal mitochondrial targeting sequence and a C-terminal cholesterol-binding STAR-related lipid transfer (START) domain [52,58,59]. While STAR is constitutively expressed under basal conditions, hormonal stimulation parallels a rapid increase in STAR levels and leads to translocation of STAR to the OMM [50,51,52]. STAR is synthesized from pre-existing mRNA, as inhibition of gene transcription did not affect induction of steroidogenesis by cAMP and only newly synthesized STAR protein is active [60,61]. Active 37 kDa STAR is rapidly processed at the OMM to an inactive 30 kDa mature protein, which is then imported to the mitochondrial matrix where it is degraded [48,62]. Active STAR has a half-life of 3–5 min in the cytoplasm, while inactive STAR has an average half-life of 2–4 h in the matrix [63,64]. Deletion of the N-terminal mitochondrial targeting sequence of STAR (N-62 STAR) resulted in no change in activity, although N-62 STAR haphazardly inserted cholesterol into other membranes, suggesting that STAR’s targeting sequence is vital for confining its activity to mitochondria [13,65]. STAR functions solely at the OMM and does not need to enter the mitochondria for its activity [66,67]. Further work has shown that the residence time of STAR at the OMM is proportional to its steroidogenic activity [68]. This activity has been shown to be tightly regulated by various mechanisms, particularly phosphorylation at Ser-194, which induces its cholesterol transfer activity by 50%, and interaction with 14-3-3γ, which negatively regulates steroid production by blocking phosphorylation of STAR at Ser-194 [60,69,70,71]. 

#### 2.1.3. STAR Function in Cholesterol Transport and the START Domain

While the exact mechanism by which STAR facilitates cholesterol transport into the mitochondria is unknown, several studies utilizing cell-free systems have shown that STAR binds cholesterol and transfers it between membranes [12,13,72]. As mentioned above, STAR contains a C-terminal cholesterol-binding START domain, a hydrophobic sterol-binding pocket composed of four α-helices and nine antiparallel β-sheets, which is common among other closely related lipid transfer proteins [59,72,73,74]. Of the START proteins, the closest homolog to STAR is STARD3, also known as metastatic axillary lymph node protein 64 (MLN64). The START domains of STAR and STARD3 have 35% sequence identity. However, while both these proteins have been shown only to bind cholesterol, STAR contains a mitochondrial targeting sequence, whereas STARD3 contains an N-terminal domain targeting the protein to late endosomes suggesting distinct functions in cholesterol transport due to differences in subcellular localization [59,75].

One current model of cholesterol transport by STAR is called the molten globule model, which has the C-helix of STAR’s START domain interacting with protonated phospholipid head groups at the OMM, inducing the C-helix to swing open [76,77]. According to this model, this conformational change allows STAR to bind and then release cholesterol [76,77]. In addition to its proposed role in binding and releasing cholesterol itself, another model suggests that STAR can trigger IMM importation of cholesterol bound to the cholesterol-binding domain of TSPO [43,76].

#### 2.1.4. Other Roles of STAR

**Figure 1:**
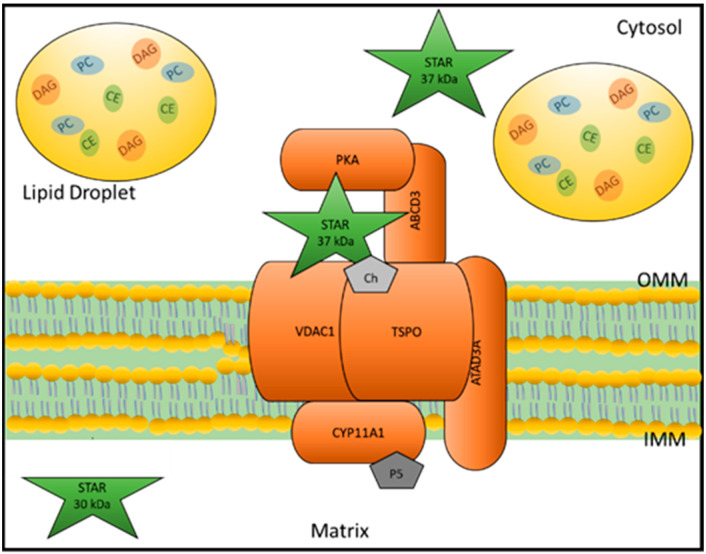
Function of hormone-induced STAR and constitutive STAR. Hormonal stimulation induces STAR to localize to the OMM where it interacts and works with other transduceosome proteins to transport cholesterol to CYP11A1 at the IMM for acute steroidogenesis. CYP11A1 converts cholesterol to the first steroid, pregnenolone. Upon reaching the OMM, the mitochondrial targeting sequence of STAR is cleaved, inactivating the protein, and inducing its import into the matrix. Constitutive STAR plays a role in the transport and/or metabolism of cholesteryl esters, diacylglycerol, and phosphatidylcholine as knockout of STAR results in the accumulation of these lipids. Abbreviations: Ch: cholesterol; P5: pregnenolone; DAG: diacylglycerol; CE: cholesteryl ester; PC: phosphatidylcholine.

Whereas most studies investigating the function of STAR have focused on the role of hormone-induced STAR in cholesterol transport for steroid biosynthesis, we developed a STAR KO MA-10 mouse tumor cell line (STARKO1) to investigate the role of constitutive STAR, i.e., STAR protein present under basal conditions independent of hormonal stimulation [10]. We showed that the absence of constitutive STAR altered lipid droplet content, specifically leading to dramatic increases in the amounts of cholesteryl ester, diacylglycerol, and phosphatidylcholine in STARKO1 cell lipid droplets [10]. Alterations in lipid droplet content paralleled alterations in the levels of many lipid-related genes. These data suggested that STAR functioned in the transport and/or metabolism of various other lipids, independent of its role in cholesterol transport for steroidogenesis. Furthermore, our recent data suggested that absence of constitutive STAR led to alterations in mitochondrial structure and function, which were exacerbated by reintroduction of STAR into STARKO1 cells [78]. Taken together these results show that STAR may have other distinct functions in addition to its known classical role in cholesterol transport for hormone-induced steroidogenesis (Figure 1).

### 2.2. SCP2/SCPx

Unlike STAR, SCP2 and SCPx have broad specificity for various lipids, and for this reason, they are referred to as non-specific lipid transfer proteins [9]. Since it is sometimes hard to tease out separate functions between the two, we use SCP2/SCPx to indicate nonspecificity. Both proteins have been shown to function in intracellular cholesterol transport between several sites such as mitochondria, ER, and plasma membrane [11,79,80]. In addition to cholesterol, previous studies have shown that SCP2/SCPx are involved in the transport and metabolism of other lipids including cholesteryl esters, fatty acids, fatty acyl-CoAs, and phospholipids [81].

#### 2.2.1. SCP2/SCPx Gene and Protein Products

The *SCP2* gene contains two distinct transcription initiation sites and encodes a 15 kDa pro-SCP2 protein and the 58 kDa SCPx, which have identical C-termini [82]. Upon further processing, the 15 kDa pro-SCP2 protein is cleaved to form the mature 13 kDa SCP2. The 58 kDa SCPx is also post-transcriptionally processed into 46 kDa SCPx, as well as the same mature 13 kDa SCP2 [83,84]. SCP2 and SCPx are both highly expressed in adrenals, testis, ovaries, liver, and intestine, all tissues with high rates of cholesterol metabolism [85]. While SCP2 and SCPx have lipid transfer activity, SCPx has been shown to be a critical enzyme in peroxisomal β-oxidation [86]. Owing to the peroxisomal targeting AKL sequence at the common C-terminus, SCP2/SCPx are both found in peroxisomes, but SCPx is exclusively localized to peroxisomes, in line with its critical function in β-oxidation, while SCP2 is also found in the cytosol [87,88,89]. Additionally, there is evidence suggesting that these proteins contain a predicted mitochondrial targeting sequence at the N-terminus, suggesting dual targeting [81,90].

#### 2.2.2. Role of SCP2/SCPx in Cholesterol Transport

Because SCP2/SCPx are both synthesized via expression of a single gene, most genetic manipulation studies described here are non-specific as to whether the findings pertain to SCP2 alone, SCPx alone, or both proteins. Several lines of evidence have supported the role of SCP2/SCPx in intracellular cholesterol trafficking. Studies have shown that recombinant human SCP2 binds cholesterol at a single binding site, however, the reported Kd values between these studies are drastically different, with one reporting a Kd of 4.2 nM and the other reporting a Kd of 0.3 μM [91,92]. Furthermore, work suggesting that SCP2/SCPx function in cholesterol transport includes in vitro studies showing that SCP2/SCPx are effective in enhancing sterol trafficking ~27-fold from plasma membranes to microsomal membranes and ~12-fold from plasma membranes to mitochondria [93]. Additionally, since cholesterol transport between lysosomes to plasma membrane occurs within two minutes in intact cells, which is inconsistent with vesicular transport, a cholesterol transport protein-mediated mechanism involving SCP2/SCPx was postulated [94]. It was shown that cholesterol transport from lysosomal membranes to plasma membranes isolated from mouse L-fibroblasts was enhanced 364-fold by SCP2/SCPx [79]. This study also showed that in L-cells plasma membranes with *Scp2* overexpressed, cholesterol levels/mg protein decreased by 38%, consistent with other data showing that SCP2 is involved in distributing cholesterol away from the plasma membrane to other cellular sites such as lipid droplets [79,95]. Additionally, cholesterol/mg protein decreased by 17% in lysosomal membranes isolated from these over-expressing L-cells, while there was a 2.2-fold increase in cholesterol/mg protein in ER membranes from the same cells [79,94]. In intact cells, transfection with human *SCP2* increased exogenous cholesterol uptake by 1.9-fold and total cholesterol mass by 1.4-fold [83]. In addition, SCP2/SCPx enhanced cholesterol transport from the plasma membrane to ER for esterification by ACAT in L-cells and enhanced intracellular cholesterol cycling in hepatoma cells [83,96,97]. SCP2 was also shown to play a role in cholesterol efflux because transfection of L-cells with *Scp2* inhibited HDL-mediated cholesterol efflux from lipid droplets to the plasma membrane through lipid rafts [95].

In addition to these studies utilizing intact cells to elucidate a role for SCP2/SCPx in cholesterol transport, studies using genetic manipulation of *Scp2* in animal models have also been done. In mice overexpressing Scp2, there was an increase in plasma LDL cholesterol, a decrease in plasma HDL cholesterol and a 70% increase in hepatic total cholesterol [98]. In *Scp2* gene-ablated mice, total hepatic cholesterol decreased 15%, likely due to decreases in cholesteryl esters [99]. In addition to these findings, animal models have also suggested a role of Scp2 in biliary cholesterol secretion. In rats with Scp2 overexpression, total hepatic cholesterol content and total bile acid content increased [98]. Conversely, in rats treated with *Scp2* antisense oligonucleotides that led to a 60% reduction in Scp2 levels in the liver, there was a delay in biliary cholesterol secretion [100]. Taken together, these data support the role of SCP2/SCPx in intracellular cholesterol transport between a variety of membranes and for a variety of critical cellular processes.

#### 2.2.3. Role of SCP2 in Steroidogenesis

In addition to these proposed functions in cholesterol trafficking by SCP2/SCPx, much work has also been done to delineate a potential role of SCP2 in cholesterol transport to the mitochondria for steroidogenesis. While STAR is known to mediate the acute, rapid transport of cholesterol from the OMM to the IMM, thereby depleting the OMM of cholesterol, many studies have been done to investigate whether SCP2 plays a role in replenishing the OMM with cholesterol from intracellular stores. In isolated rat steroidogenic adrenal cells, SCP2 enhanced radiolabeled cholesterol transport from lipid droplets to mitochondria and also significantly increased pregnenolone production [101]. Introduction of human SCP2 and the components of the cholesterol side chain cleavage system in non-steroidogenic COS-7 cells also led to increased steroid production [102]. Furthermore, human SCP2 is most highly expressed in the steroidogenic tissues (adrenals, testis, and ovaries), and hormonal stimulation of the steroidogenic cells of these tissues leads to an increase in *SCP2* mRNA expression and protein levels by a cAMP dependent pathway and increased its association with mitochondria [103,104,105,106]. Additionally, in *Scp2*-overexpressing L-cells, it was shown that sterol transport from isolated lysosomal to mitochondrial membranes was enhanced by SCP2 [107].

However, although there is much indirect evidence that SCP2 plays a role in steroidogenesis, the function of SCP2 in cholesterol transport into the mitochondria for steroid production has been called into question. Firstly, it was shown that human SCP2 enhanced cholesterol transfer to mitochondrial membranes regardless of whether the mitochondria were isolated from MA-10 cells or from fibroblasts, showing that the cells need not be steroidogenic for SCP2 to transport cholesterol to mitochondria [12]. Secondly, it was found that *Scp2* gene-ablated mice had normal serum testosterone, progesterone, and corticosteroid levels [99]. It may be the case that, although SCP2 is dispensable for steroidogenesis, it may play a role in cholesterol transport to the mitochondria as a supplementary mechanism to STAR-mediated cholesterol transport. 

#### 2.2.4. Role of SCP2/SCPx in the Transport of Other Lipids

While SCP2/SCPx are classically known as sterol transport proteins, these proteins also possess high binding affinities for many other lipid classes and have been shown to play a role in the transport and/or metabolism of many other lipids. For example, SCP2 has high affinity for fatty acids with a reported Kd of 234 nM, similar to other fatty acid binding proteins, and previous reports show that SCP2/SCPx enhance the cellular uptake and intracellular transport of fatty acids [108,109,110,111]. Further studies have shown that SCP2/SCPx function in fatty acid transport to peroxisomes for oxidation and to ER for phospholipid incorporation [111,112]. Another lipid group for which SCP2 has high affinity is fatty acyl CoAs, with reported Kds in the range of 2–4 nM [113,114]. In vitro studies and studies in intact cells have shown that SCP2/SCPx stimulate the incorporation of microsomal fatty acyl CoA into phosphatidic acid [112,115]. Additionally, SCP2 has high affinity for phosphatidylinositol (PI) and may play a role in PI transport to the plasma membrane based on data showing that human *SCP2* overexpression results in the significant redistribution of PI from mitochondrial and ER membranes to plasma membranes [116]. Lastly, SCP2 has been shown to have nanomolar affinity to all sphingolipid classes, and in vitro studies using liver homogenates suggest that SCP2/SCPx increase sphingomyelin transport [117,118,119].

#### 2.2.5. Role of SCPx in Peroxisomal β-Oxidation

**Figure 2:**
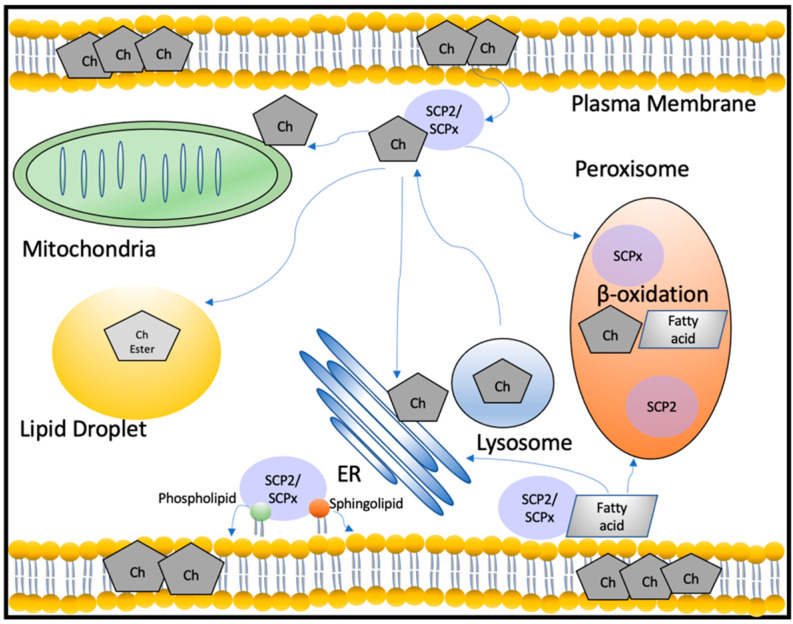
SCP2/SCPx function in the transport and metabolism of cholesterol and other lipids. SCP2/SCPx may transport cholesterol between various cellular membranes and organelles, such as the plasma membrane, mitochondria, lipid droplets, ER, lysosomes, and peroxisomes. Cholesterol intermediates also undergo oxidation in peroxisomes via SCPx for bile acid synthesis. In addition to cholesterol transport, SCP2/SCPx play a role in fatty acid, phospholipid, and sphingolipid transport. SCPx is also a key enzyme in the peroxisomal β-oxidation of fatty acids. Abbreviations: Ch: cholesterol; ER: endoplasmic reticulum.

In addition to being a lipid transporter, the 46 kDa SCPx protein that arises from 58 kDa SCPx exhibits 3-ketoacyl-CoA thiolase activity [120]. The 46 kDa SCPx is responsible for catalyzing the final step of the peroxisomal β-oxidation of branched-chain fatty acids and the metabolism of cholesterol for bile acid synthesis, while the classical 3-ketoacyl-CoA thiolase is specific for catalyzing the final step of peroxisomal β-oxidation of straight-chain fatty acids [121] [86]. Indeed, *Scp2* gene-ablated mice had defects in the metabolism of branched-chain fatty acids with a ten-fold accumulation of phytanic acid in knockout mice [99]. Further, mice null for Scpx, but with normal levels of Scp2, had altered levels of hepatic fatty acids, suggesting that branched-chain fatty acid oxidation requires SCPx independent of SCP2 [122]. The indispensable role of SCPx in peroxisomal β-oxidation was further exemplified when a homozygous 1-nucleotide insertion in *SCP2* in a patient resulted in the complete absence of SCPx protein and led to leukoencephalopathy with dystonia and motor neuropathy, hyposmia, azoospermia, and an accumulation of branched-chain fatty acids [123]. While deficiencies in several peroxisomal enzymes and/or proteins leading to neurological diseases, such as X-linked adrenoleukodystrophy and Refsum disease, have previously been reported across many patients, this was the first report of a patient with SCPx deficiency [123]. The second report of SCPx deficiency was caused by a compound heterozygous mutation in *SCP2*, again leading to undetectable levels of SCPx and neurodegenerative symptoms [124]. Recently, our laboratory worked on the characterization of a third patient with SCPx deficiency, the first associated with a heterozygous mutation in *SCP2*, leading to low, but detectable levels of SCPx [125]. Similar to previous reports, the patient presented with severe neurological symptoms. However, in contrast to previous studies, the patient’s pristanic and phytanic acid levels were normal, indicating that the patient’s low levels of SCPx were sufficient for branched-chain fatty acid metabolism. Despite normal pristanic and phytanic levels, levels of many other lipid species among various lipid classes, including fatty acids, acylcarnitines, sterols, phospholipids, and sphingolipids were altered in the patient’s fibroblasts, suggesting a role for SCPx in the transport and/or metabolism of these lipids [125]. Taken together, these data exemplify the critical and multifunctional roles of SCP2/SCPx as non-specific lipid transporters and SCPx as a key enzyme in peroxisomal oxidation (Figure 2).

## 3. Future Directions and Conclusions

Cholesterol transport proteins play vital roles in the intracellular distribution of cholesterol for several key cellular processes. Although STAR is classically known to function in cholesterol transport for hormone-induced steroidogenesis, recent studies suggest that it may have additional roles in the transport and metabolism of other lipids. While these studies point to additional roles of STAR, further studies should be done to clarify the mechanism by which STAR functions in these roles. For example, while current data supports a role of STAR in affecting lipid droplet content, it is still unclear how STAR may promote the transport of various lipids to lipid droplets. Additionally, it will be important to further investigate the role of STAR in mitochondrial structure and function since current data indicate that the absence of STAR leads to mitochondrial dysfunction. Furthermore, while SCP2/SCPx were first recognized as sterol transfer proteins, an accumulation of data ranging from cell-free systems to patients with SCP2 mutations have suggested a broader role for these proteins in lipid transport and metabolism. Additional work may also be done to further elucidate the multifunctional roles of these proteins, including the roles of these proteins in the transport and metabolism of the various lipids in distinct tissues, which has been supported by the current data. Thus, the data presented here suggest that STAR and SCP2/SCPx, classic examples of intracellular cholesterol transport proteins, play a more general role in lipid transport and metabolism in addition to their respective roles in cholesterol trafficking.
